# High Pro-Inflammatory Cytokine Secretion and Loss of High Avidity Cross-Reactive Cytotoxic T-Cells during the Course of Secondary Dengue Virus Infection

**DOI:** 10.1371/journal.pone.0001192

**Published:** 2007-12-05

**Authors:** Tao Dong, Edward Moran, Nguyen Vinh Chau, Cameron Simmons, Kerstin Luhn, Yanchun Peng, Bridget Wills, Nguyen Phuong Dung, Le Thi Thu Thao, Tran Tinh Hien, Andrew McMichael, Jeremy Farrar, Sarah Rowland-Jones

**Affiliations:** 1 MRC Human Immunology Unit, Weatherall Institute of Molecular Medicine, John Radcliffe Hospital, Oxford, United Kingdom; 2 Oxford University Clinical Research Unit, Hospital for Tropical Diseases, Ho Chi Minh City, Viet Nam; 3 Hospital for Tropical Diseases, Ho Chi Minh City, Viet Nam; Burns School of Medicine, United States of America

## Abstract

**Background:**

Dengue is one of the most important human diseases transmitted by an arthropod vector and the incidence of dengue virus infection has been increasing – over half the world's population now live in areas at risk of infection. Most infections are asymptomatic, but a subset of patients experience a potentially fatal shock syndrome characterised by plasma leakage. Severe forms of dengue are epidemiologically associated with repeated infection by more than one of the four dengue virus serotypes. Generally attributed to the phenomenon of antibody-dependent enhancement, recent observations indicate that T-cells may also influence disease phenotype.

**Methods and Findings:**

Virus-specific cytotoxic T lymphocytes (CTL) showing high level cross reactivity between dengue serotypes could be expanded from blood samples taken during the acute phase of secondary dengue infection. These could not be detected in convalescence when only CTL populations demonstrating significant serotype specificity were identified. Dengue cross-reactive CTL clones derived from these patients were of higher avidity than serotype-specific clones and produced much higher levels of both type 1 and certain type 2 cytokines, many previously implicated in dengue pathogenesis.

**Conclusion:**

Dengue serotype cross-reactive CTL clones showing high avidity for antigen produce higher levels of inflammatory cytokines than serotype-specific clones. That such cells cannot be expanded from convalescent samples suggests that they may be depleted, perhaps as a consequence of activation-induced cell death. Such high avidity cross-reactive memory CTL may produce inflammatory cytokines during the course of secondary infection, contributing to the pathogenesis of vascular leak. These cells appear to be subsequently deleted leaving a more serotype-specific memory CTL pool. Further studies are needed to relate these cellular observations to disease phenotype in a large group of patients. If confirmed they have significant implications for understanding the role of virus-specific CTL in pathogenesis of dengue disease.

## Introduction

Dengue is one of the most important human diseases transmitted by an arthropod vector (*Aedes Aegypti*) and the incidence of dengue virus infection has been increasing steadily throughout the world[1], [2]. The dengue viruses belong to the *Flavivirus* genus and there are four distinct serotypes, DEN 1 to 4. Patients develop symptoms 5–7 days after the bite of an infected mosquito. This lasts 2–7 days corresponding with the time of peak viral load. Viral titres then fall and may be low or undetectable by the day of defervescence[3]. Most infections are asymptomatic but for those with symptoms clinical manifestations range from a mild febrile illness (Dengue fever, DF) to a potentially severe syndrome which may include haemorrhage and shock (Dengue haemorrhagic fever, DHF)[4], [5].

The pathophysiology of DHF is poorly understood. The key pathological feature is increased vascular permeability with plasma leakage into the interstitial spaces associated with increased levels of vasoactive cytokines such as tumour necrosis factor alpha (TNF-α), Interferon gamma (IFN-γ), Interleukin six (IL-6) and Interleukin two (IL-2)[6]–[9]. Other implicated cytokines include IL-10, IL-13 and IL-18[10]–[12].

Epidemiological and clinical studies have demonstrated a role for immunological, host genetic and viral factors in the pathogenesis of severe disease[1]. The majority of DHF cases occur in patients who have experienced a previous infection with a heterologous DEN serotype[4], [13]. Infection with one DEN serotype generally fails to provide protective immunity against the other serotypes and sequential, heterotypic infection may lead to more severe disease. This phenomenon has been a substantial obstacle to dengue vaccine development because of the implication that cross-reactive immune responses between DEN serotypes play a part in the pathogenesis of severe disease.

The phenomenon of antibody-dependent enhancement (ADE)[14], [15] is widely accepted as a good explanation of this link between immunological cross-reactivity and disease pathogenesis. The antibody response generated against one DEN serotype provides only transient protection against other serotypes. Later infection with heterologous virus is actually enhanced by the remaining antibody with increased viral uptake into Fc receptor bearing cells. Disease severity is thought to be a consequence of the raised viral titre and the resulting pathology (whether mediated directly by the virus itself or by immune responses, cellular or otherwise) is therefore correspondingly more severe than that in primary infection. There is evidence to support the hypothesis. However ADE is neither a sufficient (estimates of rates of DHF in those experiencing secondary infection range from 1.8–12% patients[16], [17]), nor an absolutely necessary precondition for the development of severe disease (not every severe case occurs in those experiencing secondary infection – although the overwhelming majority do). Additional mechanisms are likely to be involved to account for the complex clinical phenotype of dengue disease.

It has been recognised for sometime that CTL populations are capable of mediating significant immunopathology in viral infections such as lymphocytic choriomeningitis virus[18]. There is good evidence that the CTL response to a viral infection – whether by a heterologous agent (such as one of the four dengue viruses) or one unrelated to previous viral encounters – can be modulated by the infection history of an individual in a manner likely to contribute to disease severity[19].

Recent reports have linked cross-reactive cellular immune responses to dengue virus with pathogenesis[20]–[22]. It has been proposed that cross-reactive cytotoxic memory T-cells (CTL) raised against a previous dengue strain and therefore activated in the early stages of acute secondary infection would be less efficient in eliminating newly encountered dengue virus serotypes and that this could lead to increased virus replication and more severe disease – an example of the immunological phenomenon “original antigenic sin”[21]. Others have demonstrated the complexity of the response of serotype cross-reactive memory CTL to heterologous variant peptides and suggest that cross-reactive T-cells might have altered cytokine profiles that could contribute to induction of plasma leakage[20], [23], [24].

In this study we have examined the CD8 T-cell responses to a previously identified immunodominant NS3 epitope presented by HLA-A*11[21] (the most common HLA allele in Viet Nam) in a cohort of Vietnamese dengue patients. We demonstrate a striking difference in the serotype specificity of CD8 T-cells present in patients with acute dengue virus infection compared with those in convalescence. CTL recognising tetramers for the dengue NS3 epitope can be expanded by short-term stimulation with peptides representing the dengue epitopes. Activated highly cross-reactive cells are detected in PBMCs taken during acute infection but cannot be detected in, or expanded from convalescent samples, perhaps as a consequence of activation-induced cell death *in vivo* [25], [26]. The populations remaining in convalescence show a much greater degree of serotype specificity. High avidity cross-reactive CTL clones from these patients produce higher levels of type 1 and certain type 2 cytokines than serotype specific clones. Differential release of vasoactive and pro-inflammatory factors by cross-reactive and serotype-specific CTL populations may contribute to the pathogenesis of severe dengue disease. Larger clinical studies are required to extend these observations: firstly to examine how CTL cross-reactivity and phenotype relate to disease severity and secondly to identify any differences in those experiencing primary and secondary infection. Such findings would have significant implications for dengue vaccine strategies.

## Methods

### Patient samples and HLA typing

Blood samples were collected from a cohort of over 300 adults and children attending the Hospital for Tropical Diseases and Ho Chi Minh City Children's Hospital #1 in Ho Chi Minh city, Viet Nam, with dengue virus infection of varying clinical disease severity. Samples were collected on the day of admission (acute) and during convalescence (one month after the first sample). Patients were characterised demographically, clinically (using established clinical criteria[27]), virologically (using RT-PCR) and serologically (IgM and IgG on paired samples). Serological data suggested that the majority (>99%) of the patients we studied were experiencing secondary infections. The study protocol was approved by the Scientific and Ethics committee at The Hospital for Tropical Diseases and the Oxford Tropical Research Ethics committee. Molecular HLA typing was performed on most study subjects using amplification refractory mutation system PCR (ARMS-PCR) with sequence specific primers, as previously described[28].

### Dengue virus PCR and Serology

Dengue virus RNA was isolated from acute plasma samples using RNAgents (Promega, Wisconsin). RNA was reverse transcribed and two rounds of PCR were performed using primers and methods described previously[29]. In samples containing virus, the PCR yielded DNA products, the unique sizes of which were diagnostic for each dengue serotype.

Dengue infection was confirmed via serological testing of acute and early convalescent plasma samples collected at least 3 days apart using a commercial capture-IgM and IgG ELISA (Panbio, Brisbane, Australia). The ELISA was performed and the results interpreted according to the manufacturers instructions. This ELISA assay has been validated as both sensitive and specific for primary and secondary dengue infections[30].

### Synthesis of peptide-HLA tetramers

The HLA molecule heavy chain cDNAs were modified by substitution of the transmembrane and cytosolic regions with a sequence encoding the BirA biotinylation enzyme recognition site, as previously described[31]. These modified HLA heavy chains, and ß2-microglobulin, were synthesized in a prokaryotic expression system (pET, R&D Systems), purified from bacterial inclusion bodies, solubilised in the presence of denaturant (4 M Urea) and allowed to refold with the relevant peptide by dilution. Refolded monomeric complexes were purified by FPLC and biotinylated using BirA (Avidity), then combined with either phycoerythrin (PE)-labelled, Quantum red-labelled (Sigma) or allophycocyanin (APC)-labelled streptavidin (Molecular Probes) at a 4∶1 molar ratio to form tetrameric HLA/peptide complexes (“tetramers”). Tetramers incapable of binding the CD8 molecule were produced using site-directed mutagenesis. The amino acids at positions 227 and 228 (the alpha-3 domain of the MHC molecule) were altered from DT to KA. This change abrogates CD8 binding[32]. These “CD8-null” monomers were conjugated as described above. The tetramers used in these studies were as follows: HLA-A*1101 wild type and CD8-null refolded with the previously described NS3 epitope, using three different peptide variants representing the sequences from DEN1, DEN2, and DEN3/4. Tetramers were conjugated with different fluorochromes to allow co-staining of T-cell populations.

### Antigens and antibodies

Peptides were synthesized by FMOC chemistry using a Zinnser peptide synthesiser to >70% purity. Anti-CD8 and anti-CD38 (Peridinin chlorophyll protein, PerCP) antibodies were purchased from Becton Dickinson (San Diego, California).

### Cell surface staining

Cell surface staining was carried out on freshly separated or carefully thawed cryo-preserved PBMCs. Titrated tetramers (PE, APC or quantum-red conjugated) were added for 15 min at 37°C, then the cells were incubated with CD8-APC and CD38 FITC antibodies for 15 min at room temperature (RT) in some cases. Cells were then washed and stored in Cell Fix^TM^ buffer (Becton Dickinson) at 4°C until flow cytometry analysis was performed. Samples were analyzed on a Becton Dickinson FACSCalibur.

### Tetramer dissociation assay

To assess the rate of tetramer dissociation cells from a CTL clone were incubated with PE-labelled tetramer for 40 minutes at 4°C. The cells were washed twice and resuspended in 50 µL of buffer. 2 µL of this suspension was added to 100 µL of PBS and analysed by flow cytometry. An excess of antibody known to block tetramer binding (DK25, Dako) was then added to the remaining cell suspension. 2 µL of this reaction was removed periodically, added to 200 µL of PBS and analysed on a Cyan ADP flow cytometer. The fraction of positive cells was defined as the percentage of cells falling above a gate at which 90% of cells were positive at time 0.

### Establishment of CTL lines and Clones

CTL lines were generated as previously described[33]. Briefly, PBMCs were stimulated with specific epitope peptides at 2 µM concentration, IL-2 was added on day 3, and the specificity of the CTL lines were tested using CTL lysis assay or tetramer staining on day 10 and day 20. CTL clones were established from PBMCs or CTL lines by either limiting dilution or by using the appropriate antigen-specific tetramers in combination with MACS anti-PE-microbeads (Miltenyi Biotec). Briefly, tetramers were added to 3–5 million PBMCs, then incubated at 37°C for 20 minutes. The cells were then washed with cold buffer and resuspended in 40 µl of anti-PE beads, mixed well and incubated for 15 minutes at 4–8°C. The cells were then washed and resuspended in 500 µl cold buffer, and magnetic separation was performed according to the manufacturer's protocol (Miltenyi Biotec, Germany). Sorted tetramer positive cells were counted and CTL cloning was performed by using a standard protocol as previously described[33].

### CTL lysis assays

CTL lysis assays were performed using standard ^51^Chromium release assays[33]. Briefly, HLA class I matched B-cell lines (BCL) were labelled with ^51^Chromium for 1 hour, then washed three times: target cells were then divided and pulsed with peptides at different concentrations. After another hour of incubation at 37°C, the peptide solution was then washed off and cells were counted and co-cultured with CTL clones at appropriate effector to target (E∶T) ratios in 96 well plates. The plates were incubated at 37°C for 4 hours, supernatants were harvested and then counted for radioactivity using a Beta-plate counter (WALLAC) Specific lysis was calculated from the formula:
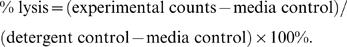



### Cytokine beads assay

In parallel with the CTL lysis assays, we performed a duplicate set of experiments using the scheme described above but without ^51^Cr labelling of the BCL. Supernatant from the CTL and target cell co-culture was harvested after overnight incubation, and cytokines were measured by Luminex cytokine bead array analysis according to the manufacturer's instructions (Bio-Rad Laboratories, USA).

## Results

### Selection of patients and peptides for the study

Twenty HLA-A*1101 positive Dengue patients were screened for responses to the previously defined HLA-A11 restricted 10-mer epitope from the dengue NS3 protein (amino acids 133–142)[21] using tetramer staining and ELISpot assays. 7 patients known to be experiencing secondary infection had epitope-specific responses detected either acutely or in convalescence and were selected for this study (Table 1). 4 of these (MD1413, BC307, MD856 and MD893) had specific responses in both acute and convalescent samples. CTL responses were studied using a panel of peptides representing the serotype variants of this epitope. The dengue 1 sequence (pD1- GTSGSPIVNR) differs significantly (by three amino acids from the C-terminal position) from the dengue 2 variant (pD2 - GTSGSPIIDK), whilst the sequences in dengue 3 and dengue 4 are identical (pD3/4 - GTSGSPIINR) and differ by only one amino acid difference at position 8. HLA-A*1101 tetramers assembled with peptides from different serotypes were labelled with different fluorochormes in order to be able to examine the ability of CD8 T-cells to recognise more than one dengue variant.

**Table 1:** Patient clinical data.

PT ID	Age	Infecting serotype	Illness severity	Days of illness
BC307	17	unknown	III	6
MD1413	10	D2	II	5
MD856	11	D4	II	5
MD893	14	D4	II	3
MD907	19	D3	DF	3
MD899	9	D4	DF	4
MD881	14	D1	DF	2

Patient identification code, age, infecting viral serotype, disease severity according to the World Health Organisation criteria (DHF grade I–IV or DF), and number of days symptoms experienced at time of acute blood sample.

### Highly cross-reactive CTL can be expanded from PBMCs in acute infection but are undetectable in convalescence

**Figure 1:**
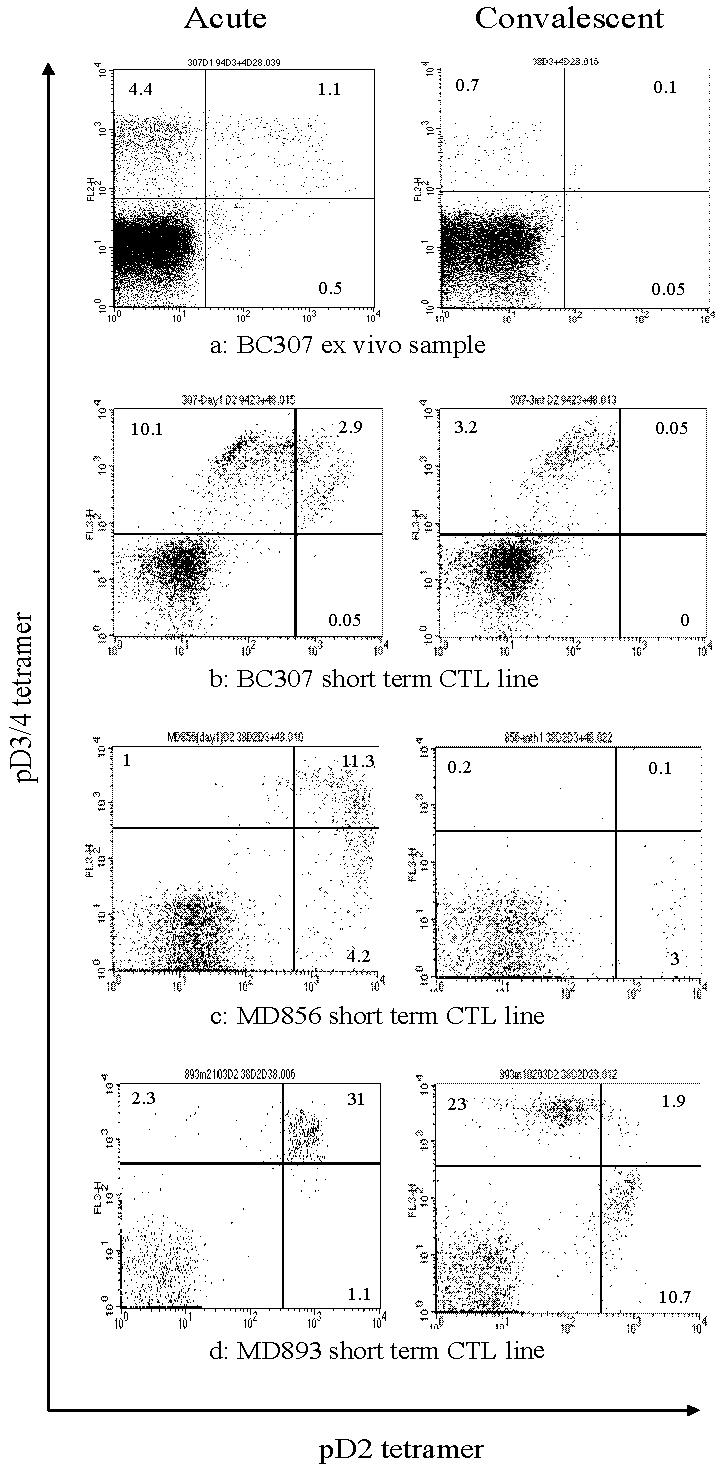
(a) Ex vivo pD2 and pD3/4 tetramer staining of PBMC from patient BC307. Freshly thawed cryopreserved PBMCs from BC307 were stained with A11 pD3/4 and pD2 tetramers and CD8 antibody. The plots are gated on CD8 positive lymphocytes. The majority of cells in the acute sample (left) are specific for pD3/4 but there is a significant population cross-reactive with this and pD2. The convalescent sample (right) shows only pD3/4 specific cells. (b,c,d) Highly cross-reactive T cells can be expanded from acute but not convalescent PBMC from dengue patients. Short term CTL lines were generated by pulsing PBMC with 2 µM of pD2 and stained with pD2 and pD3/4 tetramers on either the 14^th^ (BC307) or 20^th^ (MD856, MD893) day after stimulation. The highly cross-reactive populations apparent in the acute sample are not detectable in the convalescent sample taken one month later when more serotype specific populations have appeared.

*Ex vivo* analysis of samples taken in the acute phase of disease demonstrated great variation in the size of the dengue tetramer positive cell fraction. Patients presenting to hospital early (mostly children) had lower frequencies than those presenting later (usually adults). Although one adult patient (BC307) demonstrated large *ex vivo* tetramer positive populations acutely (Figure 1a) the majority of patients showed much less significant staining. This phenomenon may be a consequence of acute down-regulation of cell-surface TcR expression in response to the high antigen load[34]. Others have found that tetramer staining of fresh blood does not always demonstrate large numbers of virus specific CD8 T cells in acute dengue infection[21]. We observed that short-term stimulation of PBMCs with epitope peptides produced large expansions in the tetramer positive populations. Thus in order to assess the fine specificities of dengue-specific CTL with limited cell numbers, and in the face of this likely TcR down-regulation we grew short-term CTL lines by stimulating PBMCs with each of the A11 NS3 epitope variants. On day 20 after stimulation CTL were double-stained with tetramers assembled with different epitopes and labelled with different fluorochromes. At all time-points stimulation with the pD3/4 peptide resulted in the expansion of cells mostly specific for pD3/4 (data not shown). However stimulation of PBMCs from four patients (MD893, MD856, BC307, MD881) with pD2 resulted in expansions of CTL binding both D2 and D3/4 tetramers equally well. The pattern of staining differed slightly in each patient but all 4 demonstrated expansions of highly cross-reactive cells from acute samples. We considered such CTL – those recognising both pD2 and pD3/4 – to be highly cross-reactive given their equal recognition of both tetramers despite significant sequence variation. CTL recognising pD1 usually recognised pD3/4 – a reflection of their greater homology. Cells showing high cross reactivity between pD2 and pD3/4 always cross reacted with pD1. These highly cross-reactive CTL were undetectable in, and could not be expanded from the convalescent samples of patient MD893, MD856 and BC307 (Figure 1b,c,d) - no convalescent sample was available for MD881. Only CTL showing specificity for a single serotype or limited cross-reactivity (i.e. strong binding of the tetramer folded with the peptide of one serotype, weak binding of the other) remained. A similar pattern of staining was observed *ex vivo* in samples from patient BC307 where the high frequency of tetramer positive cells allowed direct analysis without the need for *in vitro* expansion. Acutely approximately 20% of tetramer positive cells were cross-reactive, recognising both the pD2 and pD3/4 tetramers. This population was not detectable in the convalescent (day 21) sample (Figure 1a).

There were 2 patients in whom this phenomenon was not observed: antigen specific cells could not be detected acutely in patient MD899 but partially cross-reactive populations recognising pD2 and pD3/4 were apparent by convalescence; patient MD907 had only serotype specific cells present acutely (pD2).

### Highly cross-reactive CTL express higher levels of CD38

**Figure 2:**
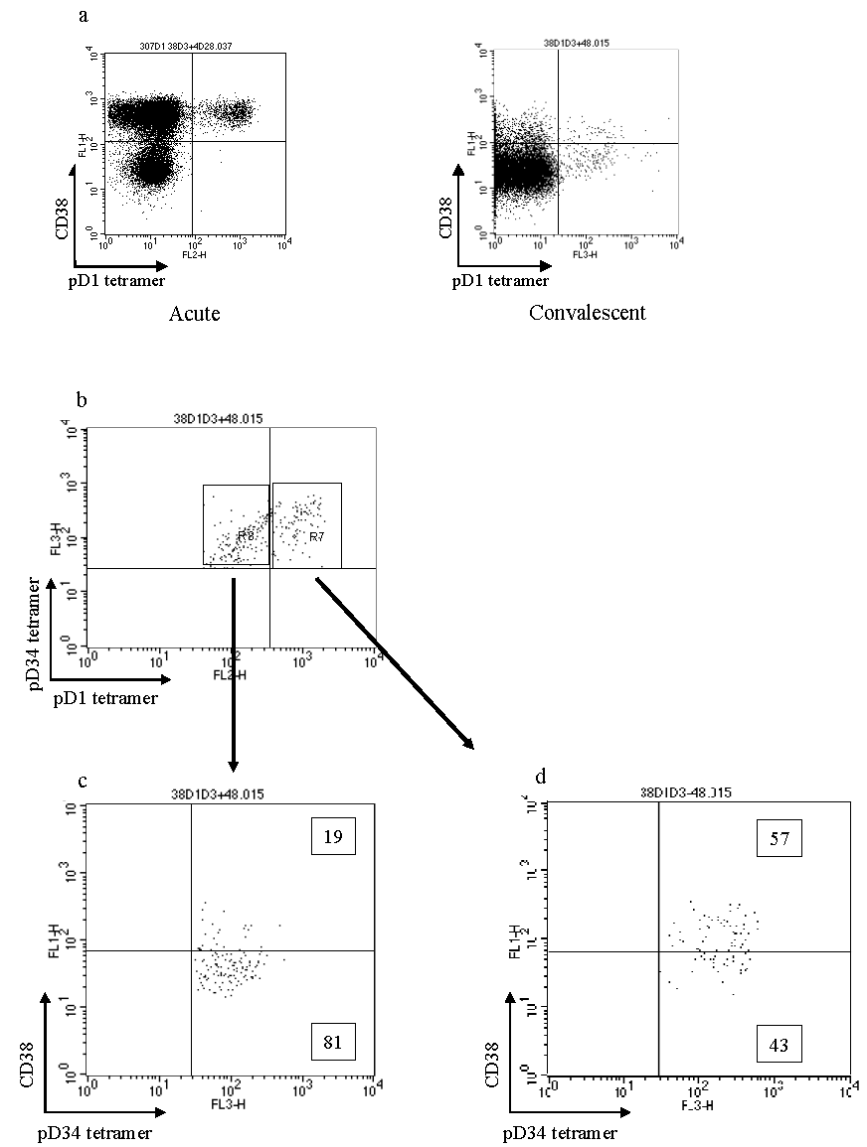
Highly cross-reactive CTL are more activated than partially cross-reactive CTL. Frozen PBMC from patient BC307 on day 21 of illness were stained with pD1 and pD3/4 tetramers together with CD38 and CD8. (a) CD38 staining of the whole CD8+ population acutely and at day 21. (b) Lymphocytes gated on CD8+ cells and co-stained with pD1 and pD3/4 tetramers. Highly cross-reactive cells (R7) show higher levels of expression of CD38 (d) than partially cross-reactive cells (c).

CD38 is a marker of lymphocyte activation. *Ex vivo* staining of PBMC from patient BC307 demonstrated equally high levels of CD38 staining among all tetramer positive cells in the acute phase (Figure 2a). By three weeks after the acute sample CD38 expression had fallen markedly. At this time point CD8 cells recognising the two similar peptide variants, pD1 and pD3/4, were present (partially cross-reactive – Figure 2b) but cells cross-reactive between the more heterologous peptides, pD2 and pD3/4 could not be detected (highly cross-reactive cells – Figure 1a). CD38 expression was greater on those T cells recognising both pD1 and pD3/4 (57% CD38 high – Figure 2d) than upon partially or non cross-reactive T cells (19% CD38 high – Figure 2c). This suggests that highly cross-reactive T cell populations are more activated than cells exhibiting low levels of cross-reactivity. It is not clear why pD1-pD3/4 cross-reactive cells are present at 3 weeks and pD2-pD3/4 cross-reactive cells are not. We could speculate that this highly cross-reactive latter group experiences even higher levels of activation resulting in activation induced cell death and clonal deletion.

### Cross-reactive CTL clones maintain cytolytic activity at low peptide concentrations

**Figure 3:**
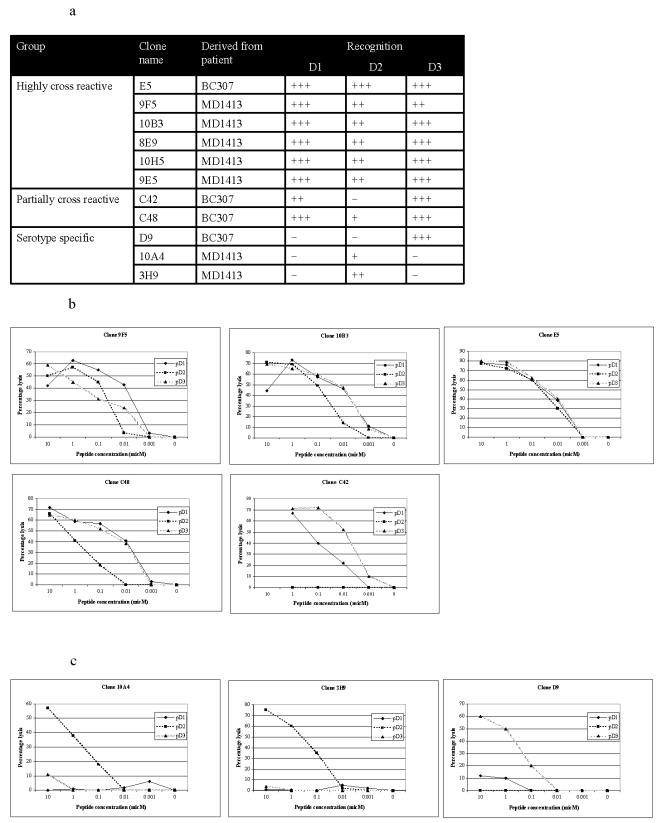
Clones differ in specificity and cytolytic efficacy in chromium-release assays. (a) Summary of clones giving the patient from whom they were generated and the variants of the dengue GTS epitope recognised by each. Peptide recognition is classified by the percentage of specific lysis of B cells loaded with 0.1 µM of peptide in a standard chromium release assay. +++ 50% or greater lysis, ++ between 20 and 50% lysis, + less than 20% lysis, − no lysis. Cross-reactive clones (b) varied in the extent to which they recognised the epitope variants on pulsed B-cells in CTL lysis assays. Serotype specific clones (c) recognising pD2 were generated from patient MD1413 and those recognising pD3/4 from patient BC307 – all showed less lytic effect at low peptide concentrations than cross-reactive clones.

We generated CTL clones by limiting dilutions of short-term lines derived from both acute and convalescent PBMC stimulated with either pD2 or pD3/4 from patients BC307 and MD1413 (Figure 3a). We were able to generate both serotype specific and highly cross-reactive CTL clones from acute samples, but only serotype-specific clones or clones showing low-level cross-reactivity could be grown from convalescent samples. Highly cross-reactive clones (E5, 10B3, 9F5, 10H5, 8E9 and 9E5) and serotype specific clones 10A4 and 3H9 (both recognising pD2) were grown from short term lines derived from acute samples. Serotype specific clones D9 (recognising pD3/4), partially cross-reactive clones C42 and C48 (both showing strongest recognition for pD3/4 and differing recognition of pD1) were grown from lines derived from convalescent samples. All highly cross-reactive clones maintained high levels of cytolytic activity at low peptide concentrations with all three peptide variants in a standard chromium release assay (9F5, 10B3 and E5 are representative – Figure 3b). Partially cross-reactive clone C48 showed good lytic activity against cells pulsed with pD3/4 and pD1 but low activity against pD2 (Fig 3b). Clone C42 showed good activity against pD3/4, low activity against pD1 and no recognition of pD2. Serotype specific clones showed intermediate (D9, 10A4 and 3H9) activity against their cognate peptides (pD3/4, pD2, pD2 respectively) but failed to recognise target cells pulsed with the peptide variants (Fig 3c). All cross-reactive or partially cross-reactive clones maintained lytic activity against B-cells pulsed with peptide concentrations as low as 0.01 µM. Serotype specific clones showed no or negligible activity at these levels.

### Cross-reactive clones show greater avidity for pD3/4 peptide-MHC

**Figure 4:**
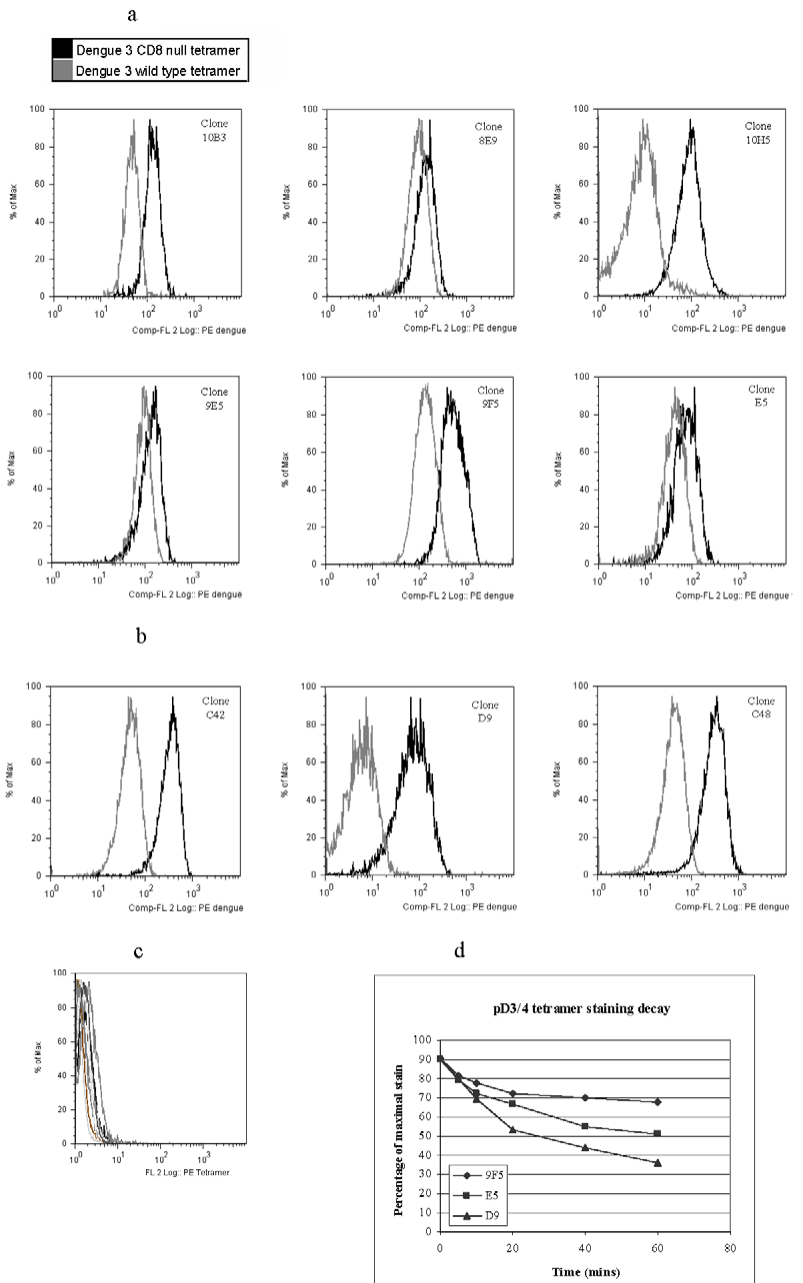
Most cross-reactive clones show significantly better staining with CD8-null tetramers than serotype-specific clones. Clones were stained with CD8-null and wild-type A11 tetramers refolded with the DEN3 variant of the GTS epitope. Cross-reactive clones (a) generally showed a smaller drop in fluorescent intensity than DEN3 specific clones which showed much poorer staining with the CD8-null tetramer than with the wild-type (b). The exception was cross-reactive clone 10H5 which showed negligible binding to pD3/4 CD8-null tetramer. No clones showed significant background staining with an unrecognised tetramer (c). pD3/4 tetramer dissociates more rapidly from serotype specific clone D9 than cross-reactive clones E5 or 9F5 (d) in a tetramer dissociation assay.

We produced tetramers incapable of binding CD8 due to a mutation in the α3 region of the heavy chain. Such tetramers have been shown to reliably identify high avidity CTLs[35]. We stained the clones with “CD8-null” tetramer folded with pD3/4. Cross-reactive clones 9F5 and E5 bound this tetramer almost as well as the wild-type tetramer (Figure 4a) whereas more serotype specific clones C48 and D9 showed little or no binding (Figure 4b). Other cross-reactive clones not described here in detail showed similarly effective binding to the CD8-null tetramer (8E9, 9E5). This implies that cross-reactive clones have a stronger peptide/MHC-TCR interaction than serotype specific clones or those with only low level cross-reactivity. The relative weakness of the peptide/MHC-TCR interaction of a serotype specific clone renders the MHC-CD8 interaction a requirement for tetramer staining. This observation was confirmed by tetramer dissociation assays. Wild-type pD3/4 tetramer dissociates more quickly from DEN3/4 serotype-specific clone D9 than either cross-reactive clone E5 or 9F5 (Figure 4d). This implies that D9's TCR avidity for tetramer is lower than its cross-reactive counterparts.

None of the clones generated from these patients showed any significant binding to the pD2 CD8-null tetramer and dissociation assays demonstrated that pD2 wild-type tetramer dissociates from cross-reactive clones much more quickly than pD3/4. pD2 conforms to published A*1101 binding motifs. It would appear that effective TCR binding is dependent upon the MHC-CD8 interaction to an extent not demonstrated by the other variants.

### Cross-reactive CTL clones produce higher levels and different patterns of cytokine release than serotype-specific CTL

**Figure 5:**
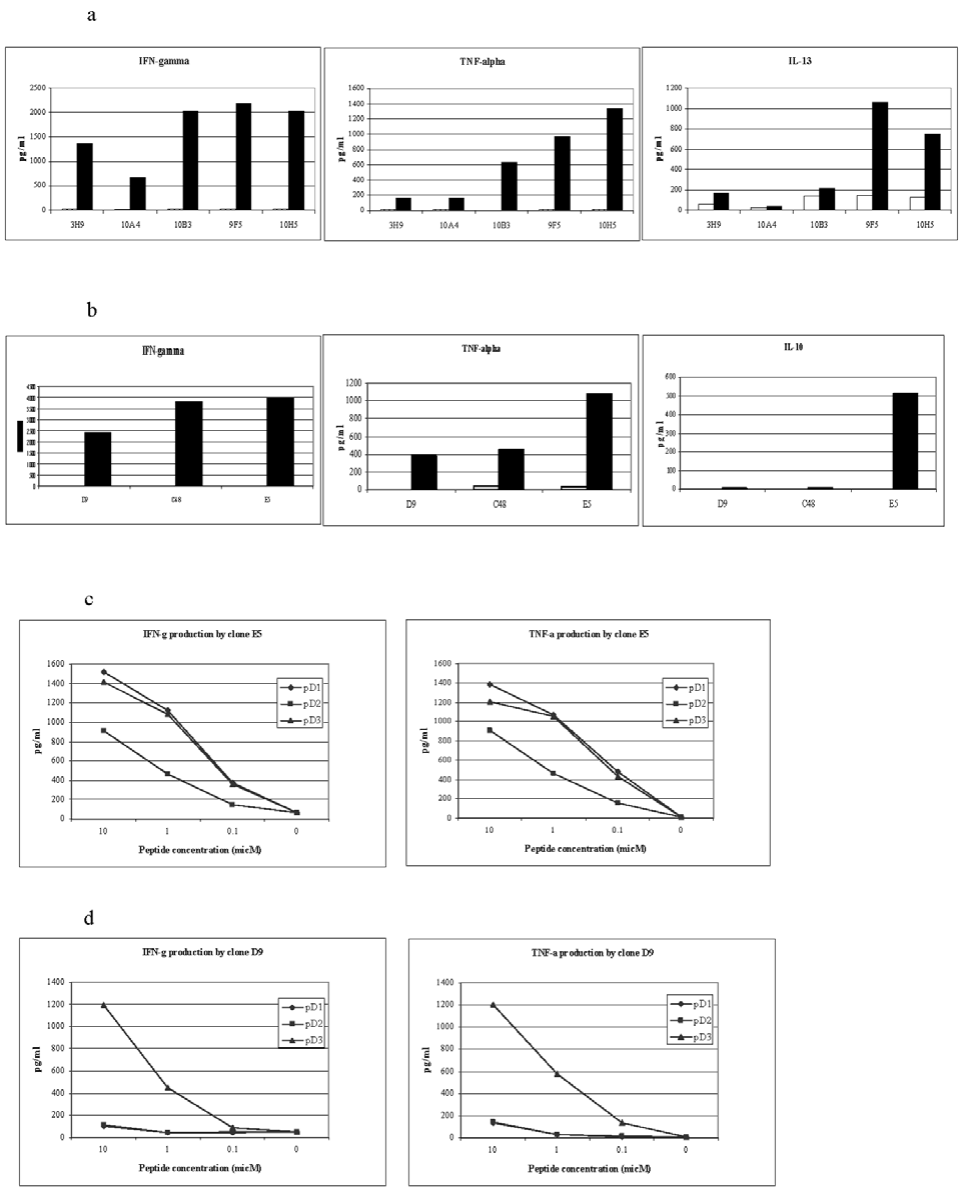
Cross-reactive clones produce higher levels of both type 1 and type 2 cytokines than serotype-specific clones. (a) Cytokines produced by cross-reactive (10B3, 9F5, 10H5) and DEN2-specific clones (3H9, 10A4) derived from patient MD1413 stimulated with B cells pulsed with 10 micM pD2 at an E∶T ratio of 5∶1. Cross-reactive clones produced slightly higher levels of each cytokine when stimulated with pD1 or pD3/4 than with pD2. Only pD2 data is shown here to allow comparison of the cross-reactive response with the DEN2 specific clones. (b) Cytokines produced by cross-reactive (E5, C48) and DEN3-specific (D9) clones derived from patient BC307 stimulated with B cells pulsed with 1 micM pD3/4 at an E∶T ratio of 10∶1. pD2 consistently resulted in a lower level of effector function than the same stimulation with pD1 or pD3/4. Black bar: peptide pulsed B cells, white bar: RPMI control pulsed B cells. Data is representative of three experiments. (c, d) IFN-γ and TNF-α production by clones E5 and D9 at an E∶T ratio of 5∶1. Both produce similar quantities when stimulated by cognate peptide at high concentrations. At lower concentrations cross-reactive clone E5 produces up to 4 times more IFN-γ than serotype specific D9. A similar phenomenon is seen with GM-CSF (data not shown). This figure is representative of three independent experiments.

Clones were stimulated with B cells loaded with the relevant peptide and the concentration of cytokines in the tissue culture supernatant measured at 24 hours. Highly cross-reactive clones E5, 9F5, 10H5 and 10B3 consistently produced higher levels of TNFα, IFNγ and GM-CSF than most serotype specific clones (Figure 5a, b). IL-10 was produced in high levels by clone E5. Other clones produced IL-10 in only very low levels if at all. The clones from patient MD1413 produced IL-13, another type 2 cytokine, and again cross-reactive clones produced significantly more. IFNγ production by the clones correlated with their cytolytic activity (compare Figure 5c/d and Figure 3). IL-4, IL-16, IL-2, and IL-1b were produced at low levels only. For many clones cytokine production was a better correlate of TCR avidity than cytolytic activity. Despite similar lytic activity against pD3/4 (Figure 3) clones E5 and C48 differ in their CD8-null binding (E5 better than C48 – Figure 4a/b) and in the cytokine production (E5 better than C48 – Figure 5a). All cross-reactive clones produced slightly less of each cytokine with pD2 stimulation. E5 is representative of this phenomenon: despite showing equally effective lytic activity to all peptide variants, it produced lower levels of IFNγ and TNFα with pD2 (Figure 5b). This is consistent with the failure to bind the pD2 CD8-null tetramer.

Cytokine production therefore reflects a clone's avidity for peptide. Cross-reactive clones tend to be of higher avidity with greater cytokine production than serotype specific clones.

## Discussion

We have demonstrated that in some hospitalised Vietnamese patients with acute dengue virus infection a population of activated high avidity CTLs showing substantial cross-reactivity between all four dengue serotypes can be detected. These high avidity cross-reactive cells cannot be detected in convalescent samples, presumably a consequence of activation-induced cell death[36], leaving only populations that show a much greater degree of serotype specificity. The phenomenon of apparent deletion of high avidity CTLs has been observed in other clinical scenarios. Molldren et al[25] studied the CTL response to the leukaemia-associated self peptide PR1, an epitope found within proteinase 3. High avidity CTL specific for PR1 were twice as effective at killing chronic myelogenous leukaemia cells than low-avidity PR1-specific CTLs[25]. These high-avidity CTLs were selectively deleted by apoptosis if exposed to high PR1 peptide concentrations or chronic myelogenous leukaemia cells overexpressing proteinase 3. Both low and high avidity PR1-specific CTLs could be detected or expanded from healthy donors, but only low avidity CTLs could be detected or expanded from newly diagnosed leukaemia patients[25]. This process of selective clonal deletion may be a result of clonal exhaustion similar to that observed in LCMV infection[37]. Certainly tetramer positive lymphocytes from patients with acute dengue show evidence of proliferating and dying in large numbers[21]. This may reflect the high avidity shown by many cross-reactive cells for their targets and the large antigen load found just before defervescence.

Tetramer positive populations are often small in acute samples stained *ex vivo*. We found that short term stimulation with pD2 produced large positive populations showing cross reactivity between pD3/4 and pD2 tetramers. In contrast stimulation with pD3/4 produced populations specific mostly for the pD3/4 tetramer in all donors with DEN3 or DEN4 infection. It has been shown that high or low avidity CTLs can be elicited *in vitro* by culturing the lymphocytes with low or high concentrations of target antigen respectively[38]–[40]. All the cross-reactive clones described here had a lower TCR avidity for pD2-MHC than either pD1-MHC or pD3/4-MHC. Thus it is possible that due to its higher avidity for the TCR the concentration of pD3/4 we used for generating CTL lines preferentially expanded lower avidity pD3/4 specific cells. In contrast although used in the same concentration as pD3/4 in the generation of short term lines, the lower avidity of pD2 for TCR resulted in the preferential expansion of the high avidity cross-reactive T-cells.

All the clones we generated in this study produced both type 1 and type 2 cytokines: IFN-γ, TNF-α, IL-13 and in some cases IL-10. Many type 1 cytokines exhibit vasoactive properties and are likely to participate in the pathogenesis of vascular leak. In addition TNF-α can mediate activation-induced cell death in some T cells [41] and has been implicated in peripheral T-cell deletion[42], [43]. Type 2 cytokines such as IL-13 and IL-10 have been implicated in the pathogenesis of severe dengue[11], [12]. IL-10 can be produced by distinct CD4+ and CD8+ T cell populations and has the ability to suppress T cell function[44], [45]. It could be postulated that certain type 2 cytokines produced in acute disease by a subset of highly cross-reactive CTL might exert an inhibitory effect on dengue specific effector T cells.

Highly cross-reactive clones grown from acute samples produced high levels of TNFα, occasionally IL-10 and demonstrated the greatest avidity for peptide-MHC as demonstrated by staining with CD8-null tetramers and tetramer decay assays. Serotype specific and partially cross-reactive clones produced much less TNF-α, IFN-γ and GM-CSF and were not shown to secrete any IL-10. It has been suggested that the pattern of CD8 cell cytokine production is epitope dependent[46], [47] with high avidity T cell/target interactions leading to greater production of TNFα or IFNγ[48]–[50].

In general killing ability correlated well with the TCR/pMHC avidity demonstrated by CD8-null tetramers. We observed variations in TCR avidity amongst clones that nonetheless displayed very similar levels of cytolytic activity for a given peptide concentration. The lytic activity of highly cross-reactive clone E5 is very similar to partially cross-reactive clone C48 for a given stimulation with pD3/4 (Figure 3). Yet E5 demonstrates much higher avidity for peptide-MHC than C48 as demonstrated by staining with CD8-null tetramers (Figure 4). The TCR/pMHC interaction is only one component, albeit perhaps the most significant, of a T cell's avidity for its target. Others include the TCR expression level, co-stimulatory molecule expression level and the extracellular microenvironment. A recent review has pointed out that T cells displaying all the characteristics of high avidity interactions may nonetheless bear a TCR that is of relatively low affinity[51]. Evidence of this complexity is apparent in our study: whereas the vast majority of cross-reactive clones producing high levels of cytokines showed high avidity TCR/pMHC interactions independent of CD8, one (10H5) did not. With that exception all cross-reactive clones showed good or moderate binding to the CD8-null tetramer but not all produced cytokines as vigorously as E5 or 9F5.

Discussion surrounds the precise roles of antibody and cellular immunity in the pathogenesis of severe dengue. It is generally accepted that antibody enhancement of secondary infection facilitates viral infection of cells leading to high viraemia, antigen loading of antigen-presenting cells[14], [15] and a consequently vigorous cellular immune response. However the observations made here suggest that not only the magnitude, but the specific “contents” of the immune response could be of significance. Memory CTL demonstrating both serotype-cross reactivity (a pre-requisite for expansion from the memory pool in secondary infection) and high avidity produce higher levels of inflammatory cytokines than their lower-avidity peers. *In vivo* this may contribute to the level of those vasoactive cytokines shown to have an association with plasma leakage and disease severity. The failure to expand these cells from convalescent samples suggests they are absent or greatly reduced in number by this time point. This may be a consequence of the large ADE-augmented antigen load combined with the high avidity of the T cells leading to over-activation, T cell exhaustion and cell death. Newly generated serotype-specific CTLs therefore dominate. It is likely that protective immunity is primarily antibody mediated, but it could be postulated that a “lack of immunopathogenesis”, as much as a degree of protection derives from these serotype-specific populations in patients with repeated exposure to dengue virus.

This detailed cellular study involved too few patients to allow firm conclusions to be drawn regarding disease aetiology. Nonetheless these findings have potentially significant implications for understanding the role of virus-specific CTL in immunity to dengue virus infection and in the pathogenesis of severe dengue disease. Further work is needed to extend them and relate the presence or absence of such high-avidity cross-reactive cells to clinical phenotype. In the hunt for a vaccine that produces pan-serotype protective immunity without the risk of iatrogenic DHF it is surely prudent to consider not only the nature of the antibody response but the specificity, avidity and effector function of T cells elicited by dengue vaccine candidates.
